# Association of ultraprocessed foods consumption and cognitive function among children aged 4–7 years: a cross-sectional data analysis

**DOI:** 10.3389/fnut.2023.1272126

**Published:** 2023-10-10

**Authors:** Shun Liu, Caimei Mo, Lidi Lei, Fangfang Lv, Jinxiu Li, Xuemei Xu, Peini Lu, Gangjie Wei, Xuanqian Huang, Xiaoyun Zeng, Xiaoqiang Qiu

**Affiliations:** ^1^Department of Child and Adolescent Health and Maternal and Child Health, School of Public Health, Guangxi Medical University, Nanning, China; ^2^Department of Epidemiology and Health Statistics, School of Public Health, Guangxi Medical University, Nanning, China

**Keywords:** ultraprocessed foods consumption, children, cognitive function, candy, cross-sectional

## Abstract

**Background:**

Sugar-sweetened beverage (SSB) consumption has shown associations with cognitive function in preschool children, but effects of other ultraprocessed foods consumption are rarely discussed in China. This study aimed to investigate the relationship between ultraprocessed food consumption and cognitive function among preschool children in China.

**Methods:**

A total of 325 children aged 4–7 years were included from Guangxi Zhuang Birth Cohort in Guangxi Zhuang Autonomous Region, China. Face-to-face interviews with parents using the Food Frequency Questionnaire (FFQ) was conducted to investigate the status of seven ultraprocessed foods consumption (i.e., chocolate, biscuits, candy, fast-food, ice cream, SSBs, and sweet bakery products). The mandarin-language version of the Wechsler Preschool and Primary Scale of Intelligence (WPPSI, Fourth Edition) was applied to assess the cognitive function of children. Multiple linear and logistic regression models were used to assess the associations between ultraprocessed food consumption and the full-scale intelligence quotient (FSIQ) and different domains and risk of cognitive deficit, respectively.

**Results:**

We found that frequent consumption of candy (β = −3.34, 95% CI: −5.62∼−1.06; *p* = 0.004) and sweet bakery products (β = −2.77, 95% CI: −5.58∼0.04; *p* = 0.054) were significant associated with decreased FSIQ scores in the linear regression models. However, only frequent consumption of candy was statistically significantly associated with an increased risk of cognitive deficit (OR = 2.05, 95% CI: 1.11∼3.79; *p* = 0.023) in the logistic regression models. For the different domains, we found frequent consumption of candy (β = −3.85, 95% CI: −6.28∼−1.43; *p* = 0.002) and sweet bakery products (β = −3.48, 95% CI: −6.47∼−0.49; *p* = 0.023) was also significantly associated with lower Verbal Comprehension Index (VCI). When combining the seven ultraprocessed foods, we found children who frequently consumed more than two kinds of ultraprocessed foods had a significant decrease of VCI scores (β = −2.66; 95% CI: −5.12∼−0.19; *p* = 0.035) too.

**Conclusion:**

Our results suggested that frequent consumption of individual (candy and sweet bakery products) and multiple ultraprocessed foods may decrease VCI scores and thereby impact cognitive function in children aged 4–7 years.

## 1. Introduction

Cognitive ability is a set of higher mental functions, including memory, learning, and attention, and is demonstrated to be a significant predictor of a child’s academic achievement (1). Globally, an estimated 200 million children under five are not reaching their potential for cognitive development (2, 3). Among them, 45 million are from China, which would place China the second largest number of children with cognitive delay in the world (4–6). Previous studies have shown that delayed cognitive development affects the academic performance and mental health of children, such as an increased risk of attention deficit hyperactivity disorder and emotional symptoms (7). Maintaining the healthy development of cognitive function has emerged as an important public health issue in children.

Childhood is a crucial period for the development of cognitive function, as the brain develops most rapidly at this stage. Brain development requires essential nutrients, implying that nutrition plays a key role in early childhood development (8, 9). A series of studies have focused on the relationship between dietary patterns, micronutrients, and cognition function in children (10–14). Among these factors, ultraprocessed food consumption, such as junk food, and snacks, especially chocolate, candy, biscuits, cake, ice creams, and salty snacks, has become of great interest to both academic research and the public (15, 16). Several studies have shown that ultraprocessed snacks, sugar-sweetened beverage (SSB), and chocolates were inversely associated with the cognitive function of children (17, 18). In addition, children with higher consumption of fast-food and SSBs at age 3 had poorer academic achievement at age 10 (19). An ultraprocessed dietary pattern with high fat, sugar, and processed food in early childhood has been associated with lower scores in verbal ability (20, 21) and increased odds of mathematical difficulties (22). Collectively, ultraprocessed food consumption may hinder the development of cognitive function in children.

In China, snacks and SSBs consumption is very common among preschool children (23–25). However, previous studies have only focused on the effects of SSB consumption on cognitive function (7, 26). To our knowledge, the effects of other ultraprocessed food consumption on children’s cognition function are rarely discussed. To address this gap in the literature, we used a cross-sectional data from the Guangxi Zhuang birth cohort in China to estimate the individual and overall effects of consuming seven common ultraprocessed foods (i.e., chocolate, biscuits, candy, fast-food, ice cream, SSB, and sweet bakery products) on cognitive function in children aged 4–7 years.

## 2. Materials and methods

### 2.1. Study participants

Study participants were from the Guangxi Zhuang Birth Cohort (GZBC), which was conducted in county-level hospitals of six major counties of the Guangxi province in China from June 2015 (27). From July to September 2021, we successfully followed up 325 children born to the mothers of GZBC in Debao and Pingguo county. During the follow-up study phase, face-to-face interviews with parents were conducted to investigate the consumption status of seven ultraprocessed foods (i.e., chocolate, biscuits, candy, fast-food, ice cream, SSB, and sweet bakery products) for the children using a Food Frequency Questionnaire (FFQ). The Mandarin-language version of the Wechsler Preschool and Primary Scale of Intelligence (WPPSI, Fourth Edition) was applied to assess the cognitive function of children. Among them, a total of 97 children were excluded which included 4 children who did not complete the food consumption survey and 93 children who did not finish the cognitive function assessment. Finally, 228 children were included for the further analysis. Ethical approval was granted by Guangxi Medical University, China (No. 20140305-001), and maternal informed consent was provided.

### 2.2. Cognitive function assessment

Children’s cognitive function was assessed using the Mandarin-language version of the Wechsler Preschool and Primary Scale of Intelligence, Fourth Edition (28), which has been widely used in cognitive function assessments because of its high reliability and validity (29). The specific cognitive function domains included Verbal Comprehension Index (VCI, a measure of the ability to understand, learn, and retain verbal information, as well as to use language to solve novel problems), Visual Spatial Index (VSI, a measure of the ability to understand visual information and to solve novel abstract visual problems), Fluid Reasoning Index (FRI, the test measures fluid intelligence, non-verbal concept formation, analysis, and problem-solving, and integration ability, etc.), Working Memory Index (WMI, a measure of the ability to hold verbal information in short-term memory and to manipulate the information), and Processing Speed Index (PSI, a measure of mental speed that may also be affected by factors such as attention) (30). The Full-Scale Intelligence Quotient (FSIQ) is a composite score of the above five domains scores (VCI, VSI, FRI, WMI, and PSI), which represents the general intellectual ability of the subject. The FSIQ and single index was standardized to have a mean of 100 and a standard deviation of 15, ranging from 40 to 160 (31). Higher values of the FSIQ or the single index represent a better test performance, and therefore stronger cognitive abilities. In addition, an FSIQ of less than 80 scores were defined as having a cognitive deficit (8, 32, 33). Regarding assessments, all examiners were trained professionally and certified before the test. The assessments were administered by well-trained examiners one-on-one, without any guidance from teachers or guardians in a standard and quiet assessment room.

### 2.3. Ultraprocessed foods assessment

Through face-to-face interviews, children’s consumption of common ultraprocessed foods in the past 3 months was determined from mothers’ responses to the Food Frequency Questionnaire (FFQ). The ultraprocessed foods in the present study included chocolate, biscuits, candy, fast-food (French fries or hamburgers), ice cream, sugar-sweetened beverage (SSB), and sweet bakery products. The food consumption frequency was reported as “never,” “once a week,” and “more than twice a week.” Finally, we defined “never” and “once a week” as “infrequent” and “more than twice a week” as “frequent” (34).

### 2.4. Covariates

Sociodemographic and family characteristics were collected via a face-to-face interview questionnaire, including child sex (male or female), child age (years), residence area (rural or urban), siblings (0. ≥ 1), parental education levels (junior high school or below, senior high school or above), annual household incomes (low = ≤ 5.99 w/year, medium = 6–14.99 w/year, high = ≥ 15 w/year), outdoor exercise time (h/day), parental accompaniment time (h/day), breastfeeding duration (months), daily sleeping time (h), and secondhand smoke (no = never, infrequent = 1–2 times/week, frequent = more than 3 times/week).

### 2.5. Statistical analysis

**FIGURE 1:**
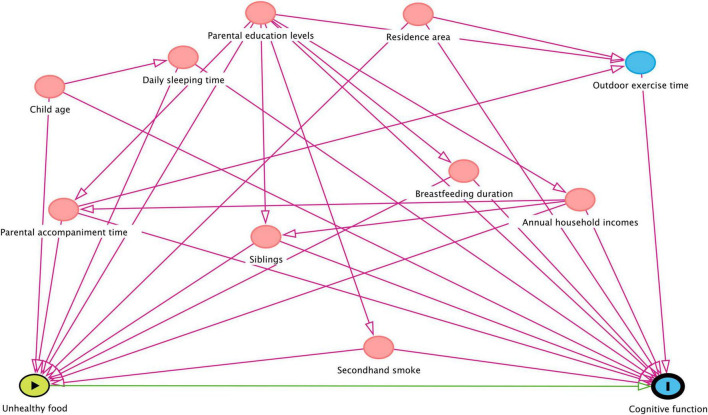
Directed acyclic graph (DAG) for the relationship of ultraprocessed foods consumption with cognitive function of children at age 4–7 years.

Categorical variables were described in frequencies and proportions, while continuous variables were described in terms of means and standardized deviations. Two variables with missing data in our analysis included daily sleeping time (18%) and secondhand smoke (7%). They were assumed to be missing at random and imputed with the multivariate imputation by chained equations (MICE) method for five times. Continuous variables were imputed using predictive mean matching, and categorical variables using logistic regression factor (2 levels) (35). The Chi-square test and Wilcoxon rank sum test was used to determine whether there were any statistically significant differences of the covariates between cognitive deficits and non-cognitive deficits. The multiple linear regression and logistic regression models were applied to analyze the relationship between ultraprocessed food consumption and cognitive function and the risk of cognitive deficit, respectively. Specifically, two models were constructed. Crude model did not adjust any factors. Adjusted model adjusted the potential confounders. A Directed Acyclic Graph^1^ was used to inspect possible pathways for confounders (36). In addition, covariates were selected *a priori* based on the previous literature (37). Finally, the following variables were selected as confounders: child age, residence area, siblings, parental education levels, annual household incomes, outdoor exercise time, parental accompaniment time, daily sleeping time, breastfeeding duration, and secondhand smoke (Figure 1). All statistical analyses were performed using R 4.03 software. The *p-*value < 0.05 were considered statistically significant. Forest plots were produced using the GraphPad Prism 8.0.1 software.

## 3. Results

### 3.1. Participants characteristics

A description of the participants (*n* = 228) characteristics is presented in Table 1. Of the 228 children, 50.88% were boys, of which, 30.70% were frequently exposed to secondhand smoke. Most of the children were from urban environments (76.75%), have siblings (73.25%), had mothers (61.40%), and fathers (62.72%) with a senior high school education or above, and less than 2 h of outdoor exercise time per day (70.18%). Among them, 45.61% had low annual household incomes. A total of 83 (36.40%) children had cognitive deficits. The distributions of residence area, siblings, parental education levels, and annual household incomes (*p* < 0.05) were significantly different between cognitive deficits and non-cognitive deficits. Especially, higher incidence of cognitive deficit was observed among children who were from urban environments, had siblings, had parents with junior high school or below education level, and had lower annual household incomes.

**TABLE 1:** Characteristics and distribution of sample children by cognitive deficit (n = 228).

Characteristics	Total (%)	Cognitive deficit		χ^2^/*z*	*P*
		**Yes (%)**	**No (%)**		
Child sex				0.376	0.540
Boy	116 (50.88)	40 (48.20)	76 (52.40)		
Girl	112 (49.12)	43 (51.80)	69 (47.60)		
Child age (year)	228	5.00 ± 0.50	4.95 ± 0.42	−0.800	0.425
Residence area	228			4.775	0.029
Rural	53 (23.25)	26 (31.30)	27 (18.60)		
Urban	175 (76.75)	57 (68.70)	118 (81.40)		
Siblings				6.510	0.011
0	61 (26.75)	14 (16.90)	47 (32.40)		
≥ 1	167 (73.25)	69 (83.10)	98 (67.60)		
Father’s education level				13.813	0.000
Junior high school or below	85 (37.28)	44 (53.00)	41 (28.30)		
Senior high school or above	143 (62.72)	39 (47.00)	104 (71.70)		
Mother’s education level				15.589	0.000
Junior high school or below	88 (38.60)	46 (55.40)	42 (29.00)		
Senior high school or above	140 (61.40)	37 (44.60)	103 (71.00)		
Annual household incomes^a^				23.084	0.000
Low	104 (45.61)	55 (66.30)	49 (33.80)		
Medium	80 (35.09)	16 (19.30)	64 (44.10)		
High	44 (19.30)	12 (14.40)	32 (22.10)		
Outdoor exercise time (h/day)				0.456	0.499
≤ 2 h/day	160 (70.18)	56 (67.50)	104 (71.70)		
> 2 h/day	68 (29.82)	27 (32.50)	41 (28.30)		
Time spent with children (father) (h/day)		7.14 ± 5.20	7.35 ± 5.64	−0.034	0.973
Time spent with children (mother) (h/day)		15 ± 7.76	13.11 ± 7.62	−1.557	0.120
Daily sleeping time (h/day)		10.31 ± 1.02	10.30 ± 0.10	−0.302	0.763
Breastfeeding duration (months)		9.77 ± 3.15	10.26 ± 3.98	−0.710	0.477
Secondhand smoke^b^				0.442	0.802
No	113 (49.56)	39 (47.00)	74 (51.00)		
Infrequent	45 (19.74)	18 (21.70)	27 (18.60)		
Frequent	70 (30.70)	26 (31.30)	44 (30.30)		

^a^Annual household incomes: Low = ≤ 5.99 w/year; Medium = 6–14.99 w/year; High = ≥ 15 w/year.

^b^Secondhand smoke: No = never, Infrequent = 1–2 times/week; Frequent = more than 3 times/week.

### 3.2. Association between frequent ultraprocessed food consumption and cognitive function among children aged 4–7 years

First, we applied a multiple linear regression model to assess the association between frequent ultraprocessed food consumption (independent variables) and FSIQ scores (dependent variables) (Table 2). In the crude model, we found children who frequently consumed candy had lower FSIQ scores compared with infrequent consumers (β = −2.49, 95% CI: −4.79∼−0.18; *p* = 0.035). After adjusting for potential confounders, we found that frequent consumption of candy (β = −3.34, 95% CI: −5.62∼−1.06; *p* = 0.004) and sweet bakery products (β = −2.77, 95% CI: −5.58∼0.04; *p* = 0.054) were significantly associated with lower FSIQ scores.

**TABLE 2:** Associations between frequent ultraprocessed foods consumption and FSIQ scores among children aged 4–7 years.

Ultraprocessed foods	Crude model^a^		Adjusted model^b^	
	**β (95% CI)**	***P***	**β (95% CI)**	***P***
Chocolate	−1.61 (−6.60∼3.38)	0.526	−1.34 (−6.36∼3.69)	0.601
Biscuits	0.32 (−2.05∼2.70)	0.788	−1.05 (−3.51∼1.41)	0.402
Candy	−2.49 (−4.79∼−0.18)	0.035	−3.34 (−5.62∼−1.06)	0.004
Fast-food	−0.83 (−6.01∼4.36)	0.753	−2.35 (−7.54∼2.84)	0.374
Ice cream	−0.51 (−2.87∼1.85)	0.668	−0.24 (−2.68∼2.19)	0.844
SSB	−0.05 (−2.73∼2.62)	0.969	−0.31 (−2.97∼2.35)	0.818
Sweet bakery products	−1.48 (−4.29∼1.33)	0.301	−2.77 (−5.58∼0.04)	0.054

FSIQ, Full-Scale Intelligence Quotient; CI, confidence interval; SSB, sugar-sweetened beverage.

^a^Crude model was not adjusted for any variables.

^b^Adjusted model was adjusted for child age, residence area, siblings, parental education levels, annual household incomes, outdoor exercise time, parental accompaniment time, daily sleeping time, breastfeeding duration, and secondhand smoke.

Second, we applied logistic regression models, including all children, to examine the association between frequent consumption of ultraprocessed foods (independent variables) and risk of cognitive deficit (dependent variables) (Table 3). In the crude model, children who frequently consumed candy showed an association with an increased risk of cognitive deficit (OR = 1.61, 95% CI: 0.94∼2.78; *p* = 0.085), whereas children who frequently consumed biscuits showed an association with a decreased risk of cognitive deficit (OR = 0.59, 95% CI: 0.34∼1.05; *p* = 0.072), both close to the level of significance. After adjusting for potential confounders, however, children who frequently consumed candy was associated with a significantly increased risk of cognitive deficit (OR = 2.05, 95% CI: 1.11∼3.79; *p* = 0.023).

**TABLE 3:** Associations between frequent ultraprocessed foods consumption and the risk of cognitive deficit among children aged 4–7 years.

Ultraprocessed foods	Crude model^a^		Adjusted model^b^	
	**OR (95% CI)**	***P***	**OR (95% CI)**	***P***
Chocolate	1.54 (0.50∼4.73)	0.455	1.13 (0.32∼3.96)	0.848
Biscuits	0.59 (0.34∼1.05)	0.072	0.78 (0.41∼1.48)	0.442
Candy	1.61 (0.94∼2.78)	0.085	2.05 (1.11∼3.79)	0.023
Fast-food	1.81 (0.56∼5.79)	0.320	2.53 (0.69∼9.22)	0.160
Ice cream	1.42 (0.82∼2.46)	0.207	1.30 (0.69∼2.42)	0.418
SSB	0.84 (0.44∼1.57)	0.578	0.84 (0.42∼1.69)	0.619
Sweet bakery products	1.02 (0.53∼1.96)	0.957	1.52 (0.72∼3.20)	0.277

OR, odds ratio; CI, confidence interval; SSB, sugar-sweetened beverage.

^a^Crude model was not adjusted for any variables.

^b^Adjusted model was adjusted for child age, residence area, siblings, parental education levels, annual household incomes, outdoor exercise time, parental accompaniment time, daily sleeping time, breastfeeding duration, and secondhand smoke.

**FIGURE 2:**
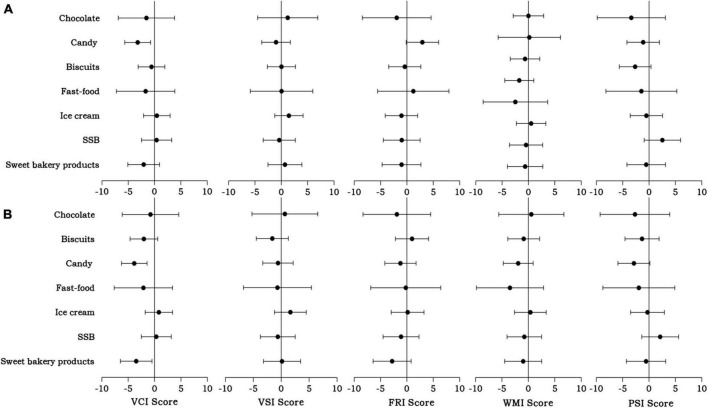
Associations between frequent consumption of ultraprocessed foods and different domain scores of the Wechsler Preschool and Primary Scale of Intelligence test among children aged 4–7 years. Crude model **(A)** was not adjusted for any variables. Adjusted model **(B)** was adjusted for child age, residence area, siblings, parental education levels, annual household incomes, outdoor exercise time, parental accompaniment time, daily sleeping time, breastfeeding duration, and secondhand smoke. SSB, sugar-sweetened beverage; VCI, Verbal Comprehension Index; VSI, Visual Spatial Index; FRI, Fluid Reasoning Index; WMI, Working Memory Index; PSI, Processing Speed Index.

The associations between frequent ultraprocessed food consumption and the specific cognitive domains, including VCI, VSI, FRI, WMI, and PSI scores, are also shown in Figure 2. In the crude model, children who frequently consumed candy showed a significant decrease in VCI scores (β = −3.20, 95% CI: −5.66∼−0.74; *p* = 0.011) while a not fully significant decrease in PSI scores (β = −2.63, 95% CI: −5.64∼−0.37; *p* = 0.085). However, children who frequently consumed biscuits showed an increase in FRI scores (β = 2.94, 95% CI: −0.14∼6.01; *p* = 0.061), which was close to the level of significance.

After adjusting for potential confounders, the associations between frequent candy consumption and lower VCI scores remained statistically significant (β = −3.85, 95% CI: −6.28∼−1.43; *p* = 0.002). In addition, a new significant association was found between lower VCI scores and frequent consumption of sweet bakery products (β = −3.48, 95% CI: −6.47∼−0.49; *p* = 0.023).

In order to analyze the combined effects of frequent consumption of the seven ultraprocessed foods on the cognitive function of children, we calculated the number (0–7) of frequent consumption of ultraprocessed foods and divided the children into frequent consumption of 0–1 and 2–7 ultraprocessed foods. Table 4 shows the multiple linear regression analysis of the associations between the numbers of ultraprocessed foods types of frequent consumption and specific cognitive function domains. In the crude models, we did not find any significant associations. However, in the adjusted models, we found that children who frequently consumed more than two kinds of ultraprocessed foods were significantly associated with decreased VCI scores (β = −2.66; 95% CI: −5.12∼−0.19; *p* = 0.035). Furthermore, we also analyzed the association between the numbers of ultraprocessed food types of frequent consumption and cognitive function and found there were no significant associations no matter FSIQ scores or risk of cognitive deficit (Table 5).

**TABLE 4:** Associations between the numbers of ultraprocessed foods types of frequent consumption and specific cognitive function domains among children aged 4–7 years.

Cognitive function domains^a^	Crude model^b^		Adjusted model^c^	
	**β (95% CI)**	***P***	**β (95% CI)**	***P***
VCI Scores	−1.77 (−4.24∼0.70)	0.160	−2.66 (−5.12∼−0.19)	0.035
VSI Scores	−0.54 (−3.19∼2.11)	0.689	−1.11 (−3.84∼1.62)	0.422
FRI Scores	0.56 (−2.47∼3.58)	0.717	−0.56 (−3.54∼2.42)	0.711
WMI Scores	−0.39 (−3.14∼2.36)	0.780	−0.76 (−3.63∼2.10)	0.600
PSI Scores	−0.05 (−3.05∼2.96)	0.975	0 (−3.07∼3.07)	1.000

VCI, Verbal Comprehension Index; VSI, Visual Spatial Index; FRI, Fluid Reasoning Index; WMI, Working Memory Index; PSI, Processing Speed Index.

^a^The results showed the effects of frequent consumption of 2–7 ultraprocessed foods compared with 0–1.

^b^Crude model was not adjusted for any variables.

^c^Adjusted model was adjusted for child age, residence area, siblings, parental education levels, annual household incomes, outdoor exercise time, parental accompaniment time, daily sleeping time, breastfeeding duration, and secondhand smoke.

**TABLE 5:** Associations between the numbers of ultraprocessed foods types of frequent consumption and cognitive function among children aged 4–7 years.

Models^a^	FSIQ scores		Risk of cognitive deficit	
	**β (95% CI)**	***P***	**OR (95% CI)**	***P***
Crude model^b^	−0.75 (−3.06∼1.57)	0.524	1.00 (0.59∼1.72)	0.990
Adjusted model^c^	−1.72 (−4.04∼0.61)	0.147	1.22 (0.66∼2.24)	0.525

FSIQ, Full-Scale Intelligence Quotient; OR, odds ratio; CI, confidence interval.

^a^The results showed the effects of frequent consumption of 2–7 ultraprocessed foods compared with 0–1.

^b^Crude model was not adjusted for any variables.

^c^Adjusted model was adjusted for child age, residence area, siblings, parental education levels, annual household incomes, outdoor exercise time, parental accompaniment time, daily sleeping time, breastfeeding duration, and secondhand smoke.

## 4. Discussion

In the present study, we employed a cross-sectional data from the Guangxi Zhuang birth cohort to assess seven common ultraprocessed foods in the cognitive function of Chinese children aged 4–7. We found children who frequently consumed candy and sweet bakery products had a significant decrease in both VCI and FSIQ scores. However, only frequent consumption of candy was significantly associated with an increased risk of cognitive deficit. Furthermore, frequently consuming more than two kinds of ultraprocessed food showed a significant association with decreased VCI scores. These findings suggested the individual and combined effects of the seven ultraprocessed foods on cognitive function among children aged 4–7 years, providing new evidence from China on the association between ultraprocessed food consumption and child cognitive development.

The ultraprocessed foods in this survey are mainly those high in saturated fat and added sugar. We showed a combined effects of these ultraprocessed food intake on children cognitive function in VCI domain, which was in line with findings of several prior epidemiology studies that ultraprocessed diet patterns with high fat and sugar content impact cognitive function (17, 18, 20, 21, 38). Animal evidence also supports the findings that these ultraprocessed food intakes may adversely influence cognitive development. For example, added sugars, especially high-fructose corn syrup, may adversely influence hippocampal function during critical periods of development in adolescent rats (39). Consuming a “Western Diet” with high saturated fat (such as chocolate, ice cream, and fast-food) has also showed adverse effects on neurocognitive function, particularly for memory processes that rely on the integrity of the hippocampus (40, 41). However, we found only frequent consumption of candy and sweet bakery products was associated with decreased cognitive scores. Results from SSB consumption were different from previous research, in which the authors suggested SSB consumption was negatively associated with executive function (26, 37). Executive function is a generic term for a range of interrelated, higher-level of cognitive abilities that are necessary for complex reasoning, goal-oriented activity, and self-regulatory behavior. The inconsistent findings may be due to the difference of population (e.g., age and lifestyles) and the assessment tools. The latest studies have shown that that candy is the most common sources of added sugar among children and adolescent (42, 43). Recently, animal studies have shown that the potentially harmful effects of long-term candy consumption on memory deficits and hippocampal neurogenesis were sufficient to reduce hippocampal levels of brain-derived neurotrophic factor (BDNF) and spatial learning performance (44). Given these finding together with the impact of candy on cognitive function, we should pay much more attention to the regulation of these food sources.

In the present study, we observed that individually the categories candy and sweet bakery products and combined effects of the seven ultraprocessed foods on cognitive function were mainly associated with decreasing VCI scores, which represented the ability to understand, learn, and retain verbal information, as well as to use language to solve novel problems. Our findings are also in line with two previous studies (20) showing that ultraprocessed dietary pattern (snack pattern) was negatively associated with lower cognitive ability, especially in verbal ability. During childhood, parenting and the family environment significantly impact verbal skills more than other performance abilities (45). Evidence shows that the prefrontal cortex and hippocampus region are critical role in verbal communication and comprehension (46, 47). Animal studies have shown that sugar could induce increases in inflammation mediators in the hippocampal (such as IL-6 and IL-1β), as well as decrease antioxidant enzymes in the frontal cortex (37). These findings lend some support to the results of the present study potentially suggesting that ultraprocessed food consumption may impact regions of the brain associated with verbal function. However, more sensitive measures such as functional magnetic resonance imaging (fMRI) techniques should be employed to assess this potential relationship further.

## 5. Strengths, limitations future research

The present study provides new evidence of the association between ultraprocessed foods consumption and cognitive performance among children aged 4–7 years. However, the following limitations should be taken into consideration when interpreting the results. First, the study relies on a cross-sectional data, which could not infer the causal relationship between ultraprocessed food consumption and cognitive development. Second, the questionnaire method in this study may cause recall bias, so we measured the frequency of consumption of ultraprocessed foods on a weekly basis, rather than monthly or yearly, mainly to reduce the influence of recall bias. Finally, assessing the cognitive performance at a single point in time may influence misclassification bias. This is because a child’s performance varies and is contingent upon the testing environment. However, in order to avoid the risk of bias as much as possible, we required testers to undergo strict training and obtain official certification. We ensured that all testers followed the evaluation procedures strictly, such as evaluating children individually in a standardized testing room. Future studies are needed to establish the causal relationship between ultraprocessed food consumption and cognitive development perhaps using more sensitive techniques such as brain imaging.

## 6. Conclusion

The results of this study suggested that the frequent consumption of the category “candy” and “sweet bakery products” and multiple ultraprocessed foods may decrease VCI scores and thereby impact cognitive function in children aged 4–7 years. It is necessary for scientists and policymakers to make targeted efforts to reduce ultraprocessed food consumption in children due to their potentially harmful effects on both physical and brain health including cognitive and emotional function.

## Data availability statement

The raw data supporting the conclusions of this article will be made available by the authors, without undue reservation.

## Ethics statement

This study was approved by the Ethical Committee of Guangxi Medical University (No.20140305–001). The studies were conducted in accordance with the local legislation and institutional requirements. Written informed consent for participation in this study was provided by the participants’ legal guardians/next of kin.

## Author contributions

SL: Data curation, Funding acquisition, Project administration, Resources, Supervision, Writing-review and editing, Writing–original draft. CM: Investigation, Methodology, Visualization, Writing–original draft. LL: Formal analysis, Methodology, Software, Writing–review and editing. JL: Supervision, Writing–review and editing. FL: Supervision, Writing–review and editing. XX: Supervision, Writing–review and editing. PL: Data curation, Supervision, Writing–review and editing. GW: Data curation, Supervision, Writing–review and editing. XH: Supervision, Writing–review and editing. XQ: Data curation, Funding acquisition, Resources, Writing–review and editing. XZ: Writing–review and editing.

## Footnotes

1
http://dagitty.net/
